# ABCG2/V-ATPase was associated with the drug resistance and tumor metastasis of esophageal squamous cancer cells

**DOI:** 10.1186/1746-1596-7-180

**Published:** 2012-12-17

**Authors:** Lijun Huang, Qiang Lu, Yong Han, Zhe Li, Zhipei Zhang, Xiaofei Li

**Affiliations:** 1 Department of Thoracic Surgery, Tangdu Hospital, The Fourth Military Medical University, Xi'an, 710038, People’s Republic of China; 2 Department of Thoracic Surgery, The Ninth Hospital of Xi'an City, Xi'an, 710054, People’s Republic of China

**Keywords:** Esophageal carcinoma, Squamous cell carcinoma, ABCG2, V-ATPase, Drug resistance

## Abstract

**Background:**

ATP-binding cassette sub-family G member 2 (ABCG2) is a protein that in humans is encoded by the ABCG2 gene. ABCG2 participates in efflux of many chemotherapeutic agents. ABCG2 is often expressed in hematopoietic progenitor or stem cells. Vacuolar-H + −ATPase (V-ATPase) plays a key role in adjusting and maintaining intracellular pH and in regulating the drug tolerance of cells. The TNM Classification of Malignant Tumours (TNM) is a cancer staging system that describes the extent of cancer in a patient’s body. In this study, the expression of ABCG2 and V-ATPase in esophageal squamous cancer cells was detected.

**Methods:**

Immunohistochemistry staining and Immunofluorescence double staining were used to detect the expression of ABCG2 and V-ATPase in in 66 cases of esophageal squamous cancer cells. Associations and differences in expression of ABCG2 with that of V-ATPase were analyzed.

**Results:**

Positive staining patterns for both ABCG2 (66.67%) and V-ATPase (68.18%) were located mainly in the plasma membrane and cytoplasm. Marked differences in expression were also shown (P < 0.001) among 3 groups of pathological grades and TNM stages in these carcinomas. Marked differences were also found for ABCG2 expression between the two groups in the pathological grades and in the TNM staging groups (P < 0.01), but not between the αb and βgroups. V-ATPase expression was statistically significant between the 2 groups in the pathological grades and TNM stages (P < 0.05). This was not evident between α and β groups of pathological grades or between αb and βof the TNM stages. Marked differences in expression of ABCG2 and V-ATPase were found between metastatic and non-metastatic groups in the same carcinomas (P < 0.0001). There was also a clear correlation between the expression of ABCG2 and V-ATPase (P ≤ 0.001) in the various groups of pathological grades and TNM stages.

**Conclusions:**

Both ABCG2 and V-ATPase were over-expressed in esophageal squamous cancer cells. Their expression was associated with pathological grade, TNM stage and tumor metastasis in esophageal squamous cancer cells, suggesting interaction relationship between them. ABCG2 and V-ATPase expression may be strongly associated with drug resistance and tumor metastasis.

**Virtual slides:**

The virtual slide(s) for this article can be found here: http://www.diagnosticpathology.diagnomx.eu/vs/3823783918433897

## Introduction

Drug efflux mediated by an ATP-binding cassette (ABC) multi-drug transporter protein is a major cause for chemotherapy treatment failure [1-3]. The second member of the transporter protein G group in ABC family, referred to as ABCG2, is often expressed in hematopoietic progenitor or stem cells. ABCG2 participates in efflux of many chemotherapeutic agents, yet many of the mechanistic functions of ABCG2 remain obscure [4,5]. In addition, vacuolar-H + −ATPase (V-ATPase) plays a key role in adjusting and maintaining intracellular pH and in regulating the drug tolerance of cells [3]. The TNM Classification of Malignant Tumours (TNM) is a cancer staging system that describes the extent of cancer in a patient’s body.

In this study, we have determined the expression of ABCG2 and V-ATPase in esophageal squamous cancer cells by immunohistochemical staining and Immunofluorescence double staining. We have further analyzed the relationship of the expression of both ABCG2 and V-ATPase with the clinical characteristics of the research study group. The key objective of this analysis was to provide clinical evidence for further study of the expression and putative drug resistance mechanisms provided by both ABCG2 and V-ATPase in esophageal squamous cancer cells.

## Materials and methods

Tissue samples were taken from 66 primary esophageal squamous cancer cells patients who had been admitted to our department for surgery from October 2006 to November 2007. The research study group of 66 patients included: 53 males and 13 females, aged between 43 to 76 years of age. The tissue samples were fixed in formalin, and following dehydration, were vitrified, and embedded in paraffin. Experts at our hospital diagnosed the pathological grade of the tissue specimens. Identification of the clinical TNM stage was based upon the invasive extent and presence or absence of distant metastases.

### Immunohistochemistry assay

The expression of ABCG2 and V-ATPase proteins in esophageal squamous cancer cells tissues was detected by immunohistochemistry according to the manufacturer's instructions. Briefly, paraffin-embedded tissues were sectioned at 4-μm and mounted on poly-L-lysine-charged glass slides. After dewaxed and rehydrated, antigen retrieval was performed by microwaving these sections in 10 mM citrate buffer (pH 6.0). To reduce nonspecific binding, slides were blocked with 100 mL.L-1 of goat serum for 30 min. Then, the sections were incubated in humidified chamber at 4°C overnight with primary anti-ABCG2(1:100, mouse IgG; Santa Cruz, San Diego, CA, USA), anti-V-ATPase (1:100, rabbit IgG; Abcam, Cambridge, MA, USA) which were diluted in 1% BSA. After the sections were washed, they were incubated with the corresponding secondary antibodies for 1 h at RT. Peroxidase activity was visualized with the DAB Elite kit (K3465, Dako), and the brown coloration of tissues represented positive staining. Finally, the sample sections were viewed by a light microscope (Zeiss Axioplan 2, Berlin, Germany).

### Immunofluorescent staining

For Immunofluorescent staining, sections were incubated with primary anti-ABCG2 antibodies (1:100, mouse IgG; Santa Cruz, San Diego, CA, USA) and anti-V-ATPase antibodies (1:100, rabbit IgG; Abcam, Cambridge, MA, USA) at 4°C overnight. After washed with PBS (5 min/wash × 3 washes), sections were incubated with Alexa Fluor® 488 Goat Anti-Mouse IgG/Alexa Fluor® 594 Goat Anti-Rabbit IgG antibodies (Invitrogen, San Diego, CA, USA) for 1 h at room temperature. The pictures were acquired by fluorescence microscopy (Zeiss Axioplan 2) and analyzed by photoshop CS2 software.

### Interpretation of immunohistochemical and immunofluorescence analyses

Protein expression of ABCG2 and V-ATPase was found to be located in the cell membrane and cytoplasm. Each sample was examined under five-high-powered fields of view under the fluorescence microscope. Data were analyzed according to a semi-quantitative evaluation. By this approach, positive cells at 5% expression were scored 0, cells with an expression of 6% - 25% were scored as 1, cells with an expression of 26% - 50% were scored as 2, cells with an expression of 51% - 75% were scored as 3, and cells with an expression >75% were scored as 4. In terms of the color of staining intensity, a yellow (Immunohistochemical) or green (*Immunofluorescence*) color was scored as 1, a clay-bank (Immunohistochemical) or red (*Immunofluorescence*) color was scored as 2, and a color of chocolate brown was scored as 3. We devised a scoring algorithm to appropriately express the differences in expression of the proteins. In this way, the approach was to multiply the scores of the relative percent positive cells with that of the staining intensity. A score of 0 was considered negative (−), scores in the range of 1–4 were considered weak positive (+), scores in the range of 5–8 were considered as intermediate positive (++), while scores in the range of 9–12 were considered as strongly positive (+++).

### Image analysis and statistical measurements

Analysis of variance was employed to analyze the data using the SPSS statistical software package (version 10.0; SPSS, Chicago, IL, USA). Three groups of data were analyzed by Kruskal-Wallis H analysis and two groups of data were analyzed by the Mann–Whitney U test. Analysis of both ABCG2 and V-ATPase, which were expressed in the same tissues, was done using Spearman’s rank correlation test and Spearman’s rank related coefficient rs. The same analytical approaches were used for the classification and expression of these proteins in the TNM staging. Statistical significance was set at an alpha value of P < 0.05.

## Results

### The expression and clinical features of ABCG2 and V-ATPase

**Figure 1:**
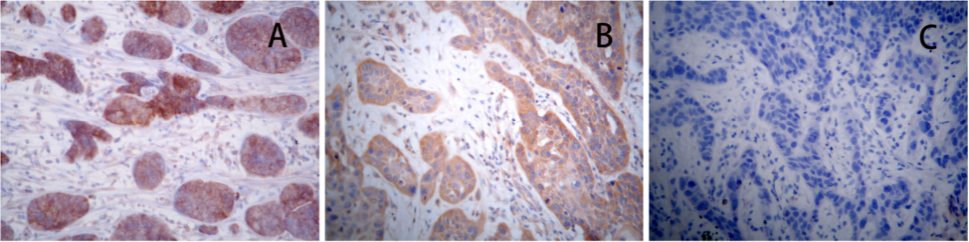
**The expression of ABCG2 and V-ATPase in esophageal squamous cancer cells. **(**A**) ABCG2 was expressed in esophageal squamous cancer cells; (**B**) V-ATPase was expressed in esophageal squamous cancer cells; (**C**) blank control. (400×).

**Figure 2:**
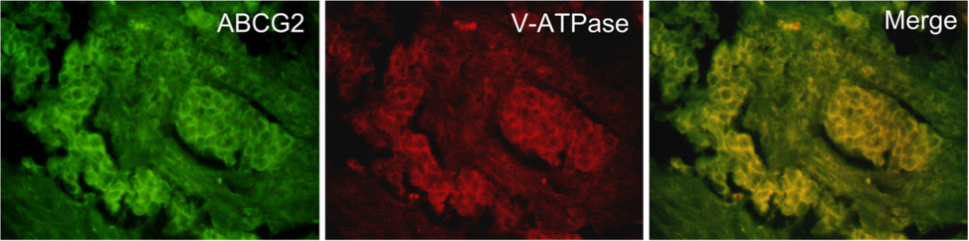
**Esophageal squamous cancer cells were detected by immunofluorescence double staining.** Immunofluorescence double staining revealed that both ABCG2 and V-ATPase were expressed on esophageal squamous cancer cells.

Immunohistochemical staining showed that expression of ABCG2 and V-ATPase were located on the cell membrane and cytoplasm (Figure 1). Immunofluorescence double staining showed that expression of both ABCG2 and V-ATPase was mainly located in the cell membrane (Figure 2). The percent positive expression of either ABCG2 or V-ATPase was respectively 65.15% (43/66) and 68.18% (45/66) in the 66 tissue samples analyzed. The expression of ABCG2 and V-ATPase depended upon the gender of the patients. In this respect, the gender-specific percent positive expression of ABCG2 was 73.58% (39/53) and 30.77% (4/13) respectively, which was also found to be statistically significant (p = 0.007), while the gender positive rates of V-ATPase were 70.36% (41/53) and 30.77%(4/13), which also had significant differences(p = 0.001). The expression of either ABCG2 (P = 0.083) or V-ATPase (P = 0.181) was not strongly associated with age.

### Differences in expression of ABCG2, pathological grading and TNM staging

ABCG2 expression was found to be significantly different among the three groups in terms of pathological and TNM staging(P < 0.0001, Table 1). In terms of the pathological grading, group I contrasted with group α and β, with a u value of 86.00 (P = 0.002) and 62.5 (P < 0.0001) respectively. Group α contrasted with group β, gave a u value of 192.0 (P < 0.005). Group α a contrasted with group αb and β were also significantly different by TNM staging, with a u value of 99.0 and 67.5 respectively (P < 0.0001). By contrast, differences between group αb compared with group β, were found not to be statistically significant with a u value of 157.0 (P = 0.341).

**Table 1:** Expression differences and clinical features of ABCG2 and V-ATPase in pathological grading and TNM staging of esophageal squamous cell carcinoma

**Group**		**Cases**	**Expression of ABCG2**					**Expression of V-ATPase**				
			**-**	**+**	**++**	**Positive rate (%)**	**Statistical value**	**-**	**+**	**++**	**Positive rate (%)**	**Statistical value**
Sex	Male	253	14	25	14	54.72	u = 188.5.0 P = 0.007	12	31	10	77.36	u = 164.0 P = 0.001
	Female	13	8	5	0	38.46		9	4	0	30.77	
Age	≤60	28	8	10	10	71.43	u = 408.0 P = 0.083	7	15	6	75.00	u = 439.0 P = 0.181
	>60	38	14	20	4	63.16		14	20	4	63.16	
pathological grading	I	15	13	0	2	13.33	${\overset{―}{\chi }}^{2}=20.36$ P = 0.000	11	2	2	26.67	${\overset{―}{\chi }}^{2}=11.89$ P = 0.003
	α	24	6	17	1	75.00		6	17	1	75.00	
	β	27	3	13	11	88.89		4	16	7	85.19	
	Total	66	22	30	14	66.67		21	35	10	68.18	
TNM staging	αa	27	18	9	0	33.33	${\overset{―}{\chi }}^{2}=26.05$ P = 0.000	17	10	0	39.26	${\overset{―}{\chi }}^{2}=22.68$ P = 0.000
	αb	22	2	14	6	90.90		2	16	4	90.90	
	β	17	2	7	8	88.23		2	9	6	87.50	
	Total	66	22	29	14	66.67		21	35	10	68.18	
Lymphatic metastasis	Yes	39	5	20	14	87.18	u = 192.5 P = 0.000	5	24	10	87.18	u = 227.0 P = 0.000
	No	27	17	10	0	37.04		16	11	0	40.74	

### Differences in expression of V-ATPase in pathological grading and TNM staging

V-ATPase expression was found to be significantly different among the three groups in terms of pathological and TNM staging (P = 0.003 and P < 0.0001 respectively, Table 1). In addition, when comparing Group I with group α and β, the u value was shown to be 109.0 (P = 0.022) and 93.0 (P < 0.002) respectively. However, group α contrasted with group β, did not demonstrate any statistically significant difference where the u value was found to be 239.5 (P = 0.06, Table 1). In addition, when comparing group αa with group α b and β, by TNM staging, the u values were found to be 117.0 and 82.0 respectively (P < 0.0001). By contrast, when comparing group αb with group β, we found no statistically significant difference, where the u value was 162.0 (P = 0.404).

### Differences in expression of ABCG2 and V-ATPase

The difference in expression of ABCG2 when contrasted with V-ATPase expression in the lymphatic metastasis group and non-lymphatic metastasis group were statistically significant (P < 0.0001, Table 1). Furthermore, the expression of ABCG2 was positively associated with the extent of V-ATPase expression. This was not only shown in esophageal squamous cancer cells tissue, but was also found to be true in the context of pathological grading and TNM staging (P < 0.001 and a correlation coefficient rs of >0.7.

## Discussion

The morbidity and mortality level of esophageal cancer in china is the highest in the world according to the report of the Chinese Centers for Cancer Registration and the Centers for Disease Control and Prevention [6]. Nevertheless, the prognosis remains relatively poor in patients with esophageal cancer, with a 5-year survival rate of 10% to 20%. Additional studies have indicated that 70% of patients already present with tumor metastases when clinical symptoms first appeared, and with a cervical lymph node metastatic rate of 73.0% to 74.5% [7]. In significant number of cases Barrett's esophagus develops to esophageal adenocarcinoma [8].

A large-scale retrospective proportional mortality study by Wang and colleagues [9] estimated that tobacco smoking was responsible for 27.9% of esophageal cancer deaths in middle-aged men and 2.8% in middle-aged women. Alcohol drinking is another important risk factor for esophageal cancer. They report separately estimates the esophageal cancer burden attributable to low intake of fruit and low intake of vegetables. Tobacco smoking, alcohol drinking, low vegetable intake and low fruit intake were responsible for 46% of esophageal cancer mortality and incidence in China. In addition, relapse and tumor metastasis were the main causes responsible for failure of surgical intervention.

Chemotherapy remains an important component of combined therapy against the relapse and metastasis of esophageal cancer [7]. However, the drug resistance or tolerance to chemotherapy displayed by esophageal carcinoma cells may be related to their comparatively low sensitivity to chemotherapy [10]. The resistance mechanisms exhibited by esophageal carcinoma cells may be associated with the following. Over-expression or enhanced functional capabilities of the ABC transport protein and second other endogenous factors that have changed during the development of the tumor. These may include altered GST (Glutathione S-transferase), which could in turn inactivate or detoxify chemotherapeuticagents, enhance DNA synthesis, or inhibit topoisomerase α activity, and change RNase activity [10,11].

The cell membrane plays a key role in tumor growth and progression [12]. The transporter of cell membrane plays a key role in pharmacology, which can functionally obstruct the absorption of chemotherapeutic agents, in which drug efflux mediated by the ATP-binding cassette (ABC) multidrug transporter protein accounts for one of the main causes of tolerance to chemotherapy [3].

ABC genes are divided into seven distinct subfamilies (ABCG2, MDR1, MRP, and so on). In recent years, research studies of the functional rolesplayed by MDR1 (Multidrug Resistance 1), MPR and ABCG2 of the ABC family have increased dramatically [13]. Moreover, ABCG2, which is the second member of group G in the ABC family is expressed highly in placental syncytial trophoblast, intestinal epithelial apical membrane, hepatic tubule membrane and brain microvascular endothelial cells. Additionally, ABCG2 plays an important role in blood–brain, blood-testis, and maternal-fetal barrier function. This transporter protein is also important in the efflux of xenobiotics, which can not only protect cells against the damage caused by extraneous substances or drugs, but it may also inhibit the anti-tumor potency or toxicological effects of chemotherapeutic drugs [14].

The membrane-associated ABCG2 consists of two distinct domains capable of undergoing conformational changes. The structure of ABCG2 consists of six reverse half-transporters, with the nuclear-binding domain at the amino-terminus, and transmembrane domain at the carboxyl-terminus capable of forming homodimers or homotrimers that can mediate the transfer or efflux of hydrophobic anions or cations, such as mitoxantrone, topotecan, doxorubicin, epirubicin, etoposide, among others [15].

There are few reports detailing ABCG2 expression in esophageal cancer. However, our study has shown that ABCG2 staining patterns in the esophageal squamous cell membrane and cytoplasm were consistent with its nature, and was located in the microsomal membrane and cytoplasm. Table 1 indicated that the total percent expression of squamous cell carcinoma was 66.7%, which was found to be significantly different among the pathological grade groups. The phenomenon that a low grade tumor has a low expression and that the high grade form of the tumor has high expression levels indicates that the expression rates of ABCG2 and its intensity of staining may be closely associated with the differentiation state of the tumor. In our study, expression of ABCG2 was also significantly different and this was dependent on TNM staging.

The epidemiology of esophageal cancer demonstrates a strong gender bias with a sex ratio of 8–9:1 in favor of males [16]. The expression of ABCG2 and V-ATPase, as we found, depended upon the gender of the patients with a sex ratio of 2.4:1 and 2.3:1 in favor of males. The expression of ABCG2 and V-ATPase was strongly associated with gender.

Our data also supported the notion that ABCG2 expression in esophageal squamous cancer cells associated with TNM staging, particularly in the context of high TNM staging and its association with high expression and enhanced positive staining intensity of ABCG2 expression. Others have shown that ABCG2 expression plays an important role in tumor stem cell proliferation, maintenance of stem cell phenotype and promotion of tumor cell development [17]. The corollary of these observations is that our data indicate that ABCG2 expression could be associated with the extent of malignancy of esophageal squamous cancer cells, the TNM staging and the metastatic features of this disease. In addition, ABCG2 expression may be associated with drug tolerance seen in esophageal squamous cancer cells. Accordingly, others have found that the drug tolerance of particular tumors is related to the intracellular and extracellular environment [18].

V-ATPase is a type of ATPase, which located on the microsomes, and expressed on the cell membrane. Multidrug resistance was related to the extracellular environment and the change of PH in cytoplasm. V-ATPase plays an important role in regulating intracellular pH [3,17]. Concordantly, we found it important to identify V-ATPase expression in esophageal squamous cancer cells. The relationship between V-ATPase activities, the relative expression of ABCG2 and the drug tolerance effect collectively exert marked influence on the functional behavior of ABCG2.

Expression of V-ATPase in esophageal squamous cancer cells, the observed pathological grading, and TNM staging informed us that overall functional expression of V-ATPase could be associated with both the pathological grading and TNM staging in esophageal squamous cancer cells. We found that the higher pathological grading and TNM staging, the higher the expression rate and more intense the positive staining for V-ATPase. For example, when comparing the lymphatic metastasis group and non-lymphatic metastasis group, expression of V-ATPase was much higher in the lymphatic metastasis group than was found in the non-lymphatic metastasis group. Since the expression of V-ATPase was closely associated with ABCG2 (Table 2, P < 0.001), in esophageal squamous cancer cells, this data indicated that it was very likely that both proteins promoted their reciprocal expression.

**Table 2:** The expression relationship between ABCG2 and V-ATPase in esophageal squamous cell carcinoma and in the pathological grading and TNM staging of esophageal squamous cell carcinoma

		**Expression of ABCG2**	**Expression of V-ATPase**			**Total**	**Statistical value**
			-	**+**	**++**		
Esophageal Squamous Cell Carcinoma		-	21	1	0	22	${\overset{―}{\chi }}^{2}=102.96$
		+	0	30	0	30	P = 0.000
		++	0	4	10	14	rs = 0.94
		Total	21	35	10	66	
pathological grading	I	-	11	2	0	13	${\overset{―}{\chi }}^{2}=15.0$
		+	0	0	0	0	P = 0.001
		++	0	0	2	2	rs = 0.76
		Total	11	2	2	15	
	α	-	6	0	0	6	${\overset{―}{\chi }}^{2}=48.0$
		+	0	17	0	17	P = 0.000
		++	0	0	1	1	rs = 1.00
		Total	6	17	1	24	
	β	-	3	0	0	3	${\overset{―}{\chi }}^{2}=32.1$
		+	1	12	0	13	P = 0.000
		++	0	4	7	11	rs = 0.80
		Total	4	16	7	27	
TNM staging	αa	-	17	1	0	18	${\overset{―}{\chi }}^{2}=20.5$
		+	0	9	0	9	P = 0.000
		++	0	0	0	0	rs = 0.856
		Total	17	10	0	27	
	αb	-	2	0	0	2	${\overset{―}{\chi }}^{2}=34.8$
		+	0	14	0	14	P = 0.000
		++	0	2	4	6	rs = 0.851
		Total	2	16	4	22	
	β	-	2	0	0	2	${\overset{―}{\chi }}^{2}=26.92$
		+	0	7	0	7	P = 0.000
		++	0	2	6	8	rs = 0.854
		Total	2	9	6	17	

It was previously shown that over-expression of V-ATPase plays an important role in maintaining the alkaline environment of the cytoplasm by regulating the cytosolic pH as a means to counter the otherwise acidic extracellular environment (15). V-ATPases were also shown to exacerbate the invasive and migratory ability of metastatic cells. Moreover, others have shown that the capacity of ABCG2 to mediate the efflux of the drug like compound topotecan, enhanced the lowering of the pH environment, and that at pH 5.5, the drug transporting ability of ABCG2 was at least five times greater than that found at physiological pH [19].

## Conclusion

Both ABCG2 and V-ATPase were highly expressed in esophageal squamous cancer cells. The pathological grading was associated with the relative ABCG2/V-ATPase expression level and staining intensity. In addition, the drug tolerance of esophageal carcinoma and the metastasis of this tumor may be associated with the expression of ABCG2/V-ATPase.

## Competing interests

The authors declare that they have no competing interests.

## Authors’ contributions

All authors contributed to this work. LH participated in the design of the study and performed the statistical analysis. QL and YH conceived of the study, participated in its design and coordination work, and helped draft the manuscript. ZL was involved in the direct clinical care (diagnosis, decision making, and treatment) of the reported patient; ZZ and XL helped search articles and revised the draft. All authors read and approved the final manuscript.
